# Exogenous glutathione protected wheat seedling from high temperature and water deficit damages

**DOI:** 10.1038/s41598-023-47868-1

**Published:** 2024-03-04

**Authors:** Mohamed Suliman Eltyeb Suliman, Safiya Babiker Mustafa Elradi, Guisheng Zhou, Tianyao Meng, Guanglong Zhu, Yunji Xu, Nimir Eltyb Ahmed Nimir, Aboagla Mohammed Ibrahim Elsiddig, Atef Hemaida Mohammed Awdelseid, Adam Yousif Adam Ali, Xiaoqian Guo, Irshad Ahmad

**Affiliations:** 1 Joint International Research Laboratory of Agriculture and Agri-Product Safety of the Ministry of Education of China, Yangzhou University, Yangzhou, 225009 China; 2 Faculty of Agriculture, University of Khartoum, 13314 Shambat, Khartoum Sudan; 3 Jiangsu Co-Innovation Center for Modern Production Technology of Grain Crops, Yangzhou University, Yangzhou, 225009 China; 4 Department of Agronomy, College of Agricultural and Environment Science, University of Al Qadarif, 32214 Al Qadarif, Sudan

**Keywords:** Abiotic, Plant physiology

## Abstract

High temperatures (HT) and drought are two major factors restricting wheat growth in the early growth stages. This study investigated the role of glutathione (GSH) amendment (0.0, 0.5, 1.0, and 2.0 mM) to soil in mitigating the adverse effect of HT (33 °C, with 25 °C as a control), water regimes (60% of field capacity and control), and their combinations. HT decreased the length, project area, surface area, volume, and forks of the root, while drought had the reverse effect. Shoot length, leaf area, leaf relative water content, and shoot and root dry matter were significantly decreased by HT and drought, and their combined impact was more noticeable. GSH significantly promoted the root system, shoot growth, and leaf relative water content. The combined treatment reduced chlorophyll *a*, chlorophyll *b*, and total chlorophyll. However, 0.5 mM GSH raised chlorophyll *a*, chlorophyll *b*, and total chlorophyll by 28.6%, 41.4%, and 32.5%, respectively, relative to 0.0 mM GSH. At combined treatment, 0.5 mM GSH decreased malondialdehyde (MDA) by 29.5% and increased soluble protein content by 24.1%. GSH meaningfully enhanced the activity of superoxide dismutase, catalase, and ascorbate peroxide in different treatments. This study suggested that GSH could protect wheat seedlings from the adverse effects of HT and/or drought stresses.

## Introduction

Wheat is one of the most important cereals as a staple food for over 1.2 billion people, ranking third in production after rice and corn^1,2^. With the growing global population, the demand for wheat is expected to increase by up to 40% by 2050 to meet food security^3^. The estimated relative yield improvement rate for wheat in numerous cropping systems has been reported to be 1%, while the demand for wheat increases by 1.7% annually, reaching a total of 1 billion tons in 2050^4^. However, wheat production is become increasingly unstable due to various abiotic stresses that occur frequently^5^.

Plants in the field are frequently subjected to abiotic stresses that severely affect their growth, development, and productivity, which results in heavy economic losses. Heat and drought are the main abiotic stresses that have detrimental impacts on wheat yield and growth, generating up to 60% and 40% yield losses globally, respectively; however, their combined impact can result in catastrophic losses^6^. Each of these stresses has been extensively studied at physiological, genetic, and molecular levels^7,8^. However, there is little research studied their combined impact on wheat plants at the early growth stage. Seedling establishment and growth are the most sensitive to heat and drought stresses at the initial stages of the growth of field crops that grow in many tropical areas^9^. Heat and drought stress negatively impact plants' growth and development and result in lower plant population^10^. Moreover, the effect will further cause physiological effects such as oxidative damage in plant cells. The photosynthetic rate in wheat plants was impaired by combination stress to a more severe level than each of the different stresses applied individually^11,12^. Combined stress can lead to high reductions in photosynthetic activity and relative leaf water content, increase accumulation of reactive oxygen species (ROS) and malondialdehyde (MDA)^10^. ROS, including hydrogen peroxide (H_2_O_2_) function as signal transduction molecules and can also cause extensive cellular damage and inhibition of photosynthesis^13^. To prevent ROS damage, the plants have developed antioxidant systems, including ROS-scavenging enzymes such as peroxidase (POD), superoxide dismutase (SOD), catalase (CAT), and ascorbate peroxide (APX). This mechanism leads to the decline of ROS compounds such as H_2_O_2_, which is eliminated by APX and CAT activities^14^. However, the antioxidant machinery of the plants can be impaired by the effect of stresses themselves^13^. Therefore, to improve plant resistance against adverse environmental conditions, numerous substances such as plant hormones have been applied.

Glutathione, known as GSH or GR, is a water-soluble non-protein compound that accounts for 1%-2% of total sulfur compounds within plant parts^15^. It is broadly distributed in almost all cell tissue and serves a wide range of biochemical functions^16^. Glutathione has high water solubility and relative stability. Because of these biochemical properties, GSH participates in a wide range of biochemical reactions^17^. Thus, it can provide stress protection in various ways. In addition to the direct role in stress injury protection, GSH can also modulate some other antioxidants. It acts as a co-factor, interacts with hormones and redox molecules, and participates in stress-induced signal transduction^18,19^.

Exogenous GSH improved the activities of SOD, CAT, POD, and other enzymes correlated to the AsA-GSH cycle, including APX, DHAR, MDHAR, and GR, and declined the content of H_2_O_2_ and superoxide anion (O_2_^-^), and lipid peroxidation levels^20^. Application of GSH significantly mitigated drought stress-induced oxidative damage in mung bean seedlings by improving the capacity of glyoxalase and antioxidant systems^21^. Also, GSH increased plant growth, chlorophyll content, and photosynthetic rates in cucumber (*Cucumis sativus* L.) seedlings under high temperature stress. The improvement in stressed-seedling is attributed to regulating morphological, physiological, and biochemical parameters^22^. Furthermore, GSH enhanced salinity stress tolerance at seedling and reproductive stages in the soybean (*Glycine max*) according to^23^. They reported that exogenous GSH improved plant height, number of branches per plant, number of pods per plant, seeds per pod, seeds per plant, and 100-seed weight, which sequentially increased plant productivity. At the seedling stage, GSH improved the oxidative stress tolerance as indicated by lower H_2_O_2_ and MDA levels.

The present study attempted to elucidate the role of exogenous GSH in alleviating oxidative stress induced by the independent and combined effects of heat and drought stress in wheat seedlings. The impacts of these stresses were studied on growth, dry matter, chlorophyll pigments, lipid peroxidation by MDA, total soluble protein, and antioxidant enzyme activity.

## Results

### Root

Analysis of variance showed that temperature, drought, and their combinations with GSH had diverse effects on different parameters of wheat root (Tables 1 and 2). High temperature (HT) stress decreased the length, project area, surface area, diameter, volume, tips, forks, fresh, and dry weight of the root by 29.5%, 44.0%, 51.7%, 19.4%, 49.2%, 12.1%, 43.5%, 50.5%, and 28.1%, respectively, compared with control. However, drought (Wd) increased the length, project area, surface area, volume, tips, and forks of the root by 24.0%, 31.5%, 22.5%, 34.5%, 46.5%, and 44.0%, respectively, relative to control. Moreover, Wd reduced the diameter and fresh and dry weight of the root by 36.8% 16.1% and 20.3%, respectively, relative to Wn. The interaction effect of HT and Wd declined the length, project area, surface area, volume, and fresh weight of the root by 12.9%, 27.4%, 42.8%, 32.4%, and 62.0%, respectively. While root tips were increased by 69.3% as compared with 25 °C and Wn. Application of GSH significantly enhanced most of the measured root growth parameters. The application of 0.5 mM GSH, increased the project area, surface area, diameter, volume, and fresh weight of the root by 31.5%, 22.4%, 59.8%, 38.2%, and 23.8%, respectively, compared with 0.0 mM of GSH. In addition, root tips were increased gradually with increasing hormone dosages. 2.0 mM GSH raised root tips by 18.5% in comparison to 0.0 mM GSH.

**Table 1:** The ANOVA for roots attributes of wheat plants as influenced by water regime, temperature, and 5-ALA application.

Source of variation	Root length	Project area	Surface Area	Root diameter	Root volume	Tips	Forks	Root fresh weight	Root dry weight	Shoot length	Leaf area	Shoot fresh weight	Shoot dry weight
	F Value												
Temperature (T)	38.48*	25.78*	65.6*	1.19^ns^	26.89*	2.09^ns^	25.25*	88.13*	16.58*	9.84*	117.88*	422.82*	5.98*
Drought (D)	18.75*	6.29*	5.55*	5.17*	5.39*	22.97*	10.63*	5.59*	7.90*	17.16*	14.01*	48.37*	0.04^ns^
GSH	0.30^ns^	1.22*	0.97*	1.11^ns^	1.25*	0.91^ ns^	0.33^ns^	1.62*	0.56^ns^	7.10*	6.20*	7.11*	0.69*
T × D	4.52*	2.54*	4.57*	0.13*	1.19*	1.28*	0.51*	1.39*	0.07*	4.96*	0.21*	0.80*	0.86^ns^
T × GSH	0.95^ns^	0.63^ns^	0.79^ns^	0.01^ns^	0.25^ns^	0.41^ns^	0.44^ns^	1.54^ns^	0.60^ns^	0.44^ns^	2.41^ns^	2.19^ns^	0.9^ns^
D × GSH	0.13^ns^	0.15^ns^	0.48^ns^	0.48^ns^	0.29^ns^	0.28^ns^	0.60^ns^	0.99^ns^	0.41^ns^	1.18^ns^	0.65^ns^	1.70^ns^	0.8^ns^
D × T × GSH	0.25^ns^	0.01^ns^	0.01^ns^	0.01^ns^	0.05^ns^	0.66^ns^	0.17^ns^	0.24^ns^	0.07^ns^	1.33^ns^	117.88^ns^	0.61^ns^	5.9^ns^

^ns^, * indicate statistical non-significance or significance at the 0.05 probability level, respectively.

**Table 2:** Effect of temperature, drought, and GSH on root parameters of wheat seedling.

Temperature (T) °C	Root length	Project area	Surface area	Root diameter	Root volume	Tips	Forks	Root fresh weight	Root dry weight
	(cm)	(cm^2^)	(cm^2^)	(mm)	(mm^3^)			(mg)	(mg)
25	138.01a	9.41a	21.78a	0.845a	0.447a	713.68b	884.94a	261.64a	18.58a
33	97.34b	5.27b	10.51b	0.681b	0.227b	799.95a	499.95b	128.71b	13.36b
Water regime (W)									
W_n_	105.07b	6.34b	14.51b	0.935a	0.287b	613.93b	567.58b	212.23a	17.77a
W_d_	130.28a	8.34a	17.78a	0.591b	0.386a	899.70a	817.34a	178.13b	14.17b
T × W									
25 × W_n_	119.70ab	7.79b	18.65a	0.990^ ns^	0.374ab	537.11b	732.73^ ns^	269.98a	20.56^ ns^
25 × W_d_	156.33a	11.03a	24.90a	0.701	0.520a	890.25a	1037.16	253.30a	16.61
33 × W_n_	90.44b	4.89c	10.36b	0.879	0.201b	690.75ab	402.43	154.48b	14.99
33 × W_d_	104.23b	5.65bc	10.66b	0.482	0.253ab	909.15a	597.48	102.95b	11.73
GSH (mM)									
0.0	112.92^ ns^	6.05b	14.26b	0.525b	0.267b	676.08b	670.82^ ns^	169.43b	14.83^ ns^
0.5	122.18	7.95a	17.45a	0.844a	0.369a	758.53a	699.46	209.68a	16.77
1.0	118.69	7.77a	16.73a	0.844a	0.360a	791.60a	649.18	203.40a	15.52
2.0	116.91	7.59a	16.14a	0.839a	0.351a	801.05a	750.33	199.87a	16.76

W_n_: Normal watering; W_d_: drought. The means followed by different letters in the same column are statistically different at the 0.05 probability level. ^ns^ is not statistically significant between means in the same column.

### Shoot

Data in Tables 1 and 3 showed that temperature, drought, and their combinations with GSH had various effects on the shoot parameters of wheat. HT, water deficit, and their combination significantly reduced all the measured shoot parameters as compared with control, with the highest reductions in the combination treatment. The temperature of 33 °C and Wd decreased shoot length, leaf area, shoot fresh weight, and leaf water relative content by 34.8%, 75.5%, 63.5%, and 16.7%, respectively, compared with 25 °C and Wn. Exogenous application of GSH significantly improved shoot parameters. 2.0 mM GSH increased shoot length and leaf area by 10.0% and 67.5%, respectively, compared with 0.0 mM GSH. The application of 0.5 mM GSH raised fresh shoot weight by 24.7%, while 1.0 mM increased leaf water relative content by 9.8% in relation to 0.0 mM GSH.

**Table 3:** Effect of temperature, drought, and, GSH on shoot parameters of wheat seedling.

Temperature (T) °C	Shoot length	Leaf area	Shoot fresh weight	Shoot dry weight	Relative water content
	(cm)	(cm^2^)	(mg)	(mg)	(%)
25	25.28a	6.26a	207.09a	23.99a	82.96a
33	18.39b	2.32b	99.29b	15.78b	74.83b
Water regime (W)					
W_n_	22.87a	4.97a	171.47a	22.25a	81.93a
W_d_	20.80b	3.61b	134.92b	17.52b	75.89b
T × W					
25 × W_n_	25.76a	7.03a	227.65a	26.63^ns^	85.29a
33 × W_d_	24.80a	5.50a	186.54b	21.35	80.64ab
25 × W_n_	19.98b	2.91b	115.28c	17.87	78.57ab
33 × W_d_	16.80c	1.72b	83.00c	13.68	71.08b
GSH (mM)					
0.0	20.98c	2.95b	135.28c	18.16^ns^	73.89b
0.5	21.70b	4.68a	168.70a	21.63	80.21a
1.0	22.53ab	4.58a	151.32b	19.91	81.11a
2.0	23.08a	4.95a	157.47b	19.84	80.36a

W_n_: Normal watering; W_d_: drought. The means followed by different letters in the same column are statistically different at the 0.05 probability level. ^ns^ is not significant between means in the same column.

### Chlorophyll content

**Figure 1:**
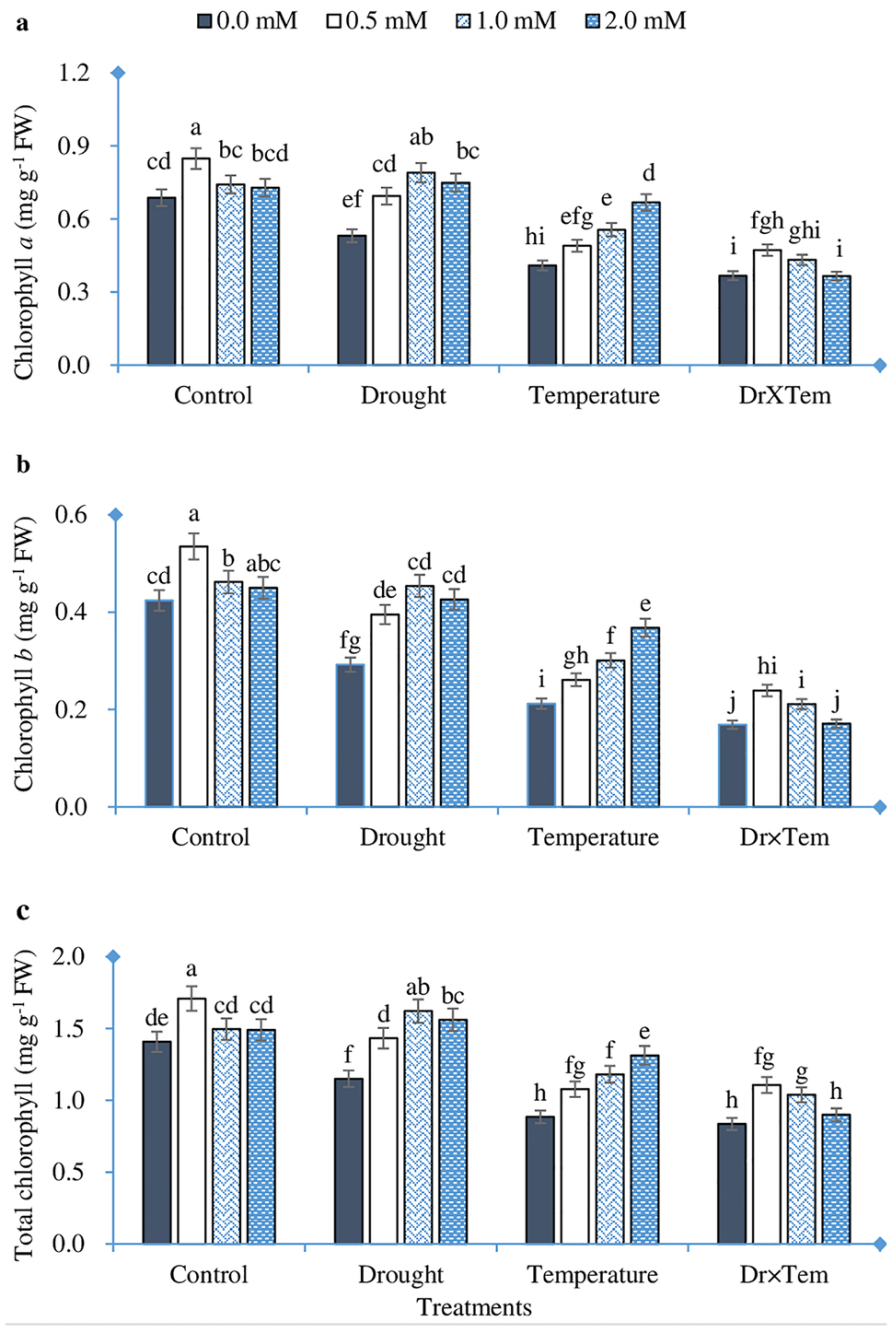
Effect of high temperature, drought, GSH, and their interactions on chlorophyll contents of wheat seedlings. (**a**) Chlorophyll *a*, (**b**) chlorophyll *b*, (**c**) total chlorophyll. Bars labeled with different letters indicate significant difference at P < 0.05.

The effects of drought, temperature, GSH, and their interactions on chlorophyll parameters can be seen in Table 4 and Fig. 1. Wd decreased the content of chlorophyll a, chlorophyll b, and total chlorophyll by 22.7%, 31.1%, and 18.3%, respectively, as compared with Wn. The same parameters were declined by HT stress compared to drought stress. Whereas the combined effect greatly lowered chlorophyll parameters than the individual effect. The combination of Wn and HT reduced the content of chlorophyll a, chlorophyll b, and total chlorophyll by 46.6%, 60.1%, and 40.7%, respectively, as compared with the control. Exogenous application of GSH at different levels had various positive impacts on chlorophyll content. In this regard, 0.5 mM GSH achieved higher chlorophyll content in the control and combined treatments. Though 1.0 and 2.0 mM GSH had the greatest chlorophyll content in the drought and temperature treatments, respectively. In the control treatment, 0.5 mM GSH increased the content of chlorophyll a, chlorophyll b, and total chlorophyll by 23.4%, 26.2%, and 21.4%, relative to 0.0 mM GSH. Also, in the combined treatment, 0.5 mM GSH raised the content of chlorophyll a, chlorophyll b, and total chlorophyll by 28.6%, 41.4%, and 32.5%, respectively, in comparison to 0.0 mM GSH.

**Table 4:** The ANOVA for shoot and physiological attributes of wheat plants as influenced by water regime, temperature, and GSH application.

Source of variation	Relative water content	Chlorophyll *a*	Chlorophyll *b*	Total chlorophyll	SOD	CAT	Protein	MDA	APX
	F Value								
Temperature(T)	29.33*	42.21*	36.48*	28.44*	5.65*	5.72*	631.82*	58.43*	4.56*
Water regime (W)	14.01*	8.60*	6.86*	3.56*	1.71^ ns^	2.85*	166.98*	7.36*	0.13^ ns^
GSH	4.88*	4.44*	3.98*	4.49*	2.94*	1.57*	20.81*	1.62*	3.81*
T × W	0.01*	2.11^ ns^	0.06^ ns^	2.58^ ns^	0.03*	0.23*	56.12*	2.61*	1.37^ ns^
T × GSH	1.09^ ns^	0.56^ ns^	0.47^ ns^	0.09^ ns^	0.16*	0.01^ ns^	14.31*	0.47^ ns^	3.18*
W × GSH	0.17^ ns^	0.14^ ns^	0.26^ ns^	0.41^ ns^	0.41^ ns^	0.24*	2.73^ ns^	0.17*	0.82^ ns^
T × W × GSH	0.07^ ns^	1.04*	1.31*	1.36*	0.06*	0.03*	0.56*	0.38*	2.99*

^ns^, * indicate statistical non-significance or significance at the 0.05 probability level, respectively.

### Malondialdehyde and soluble protein contents

**Figure 2:**
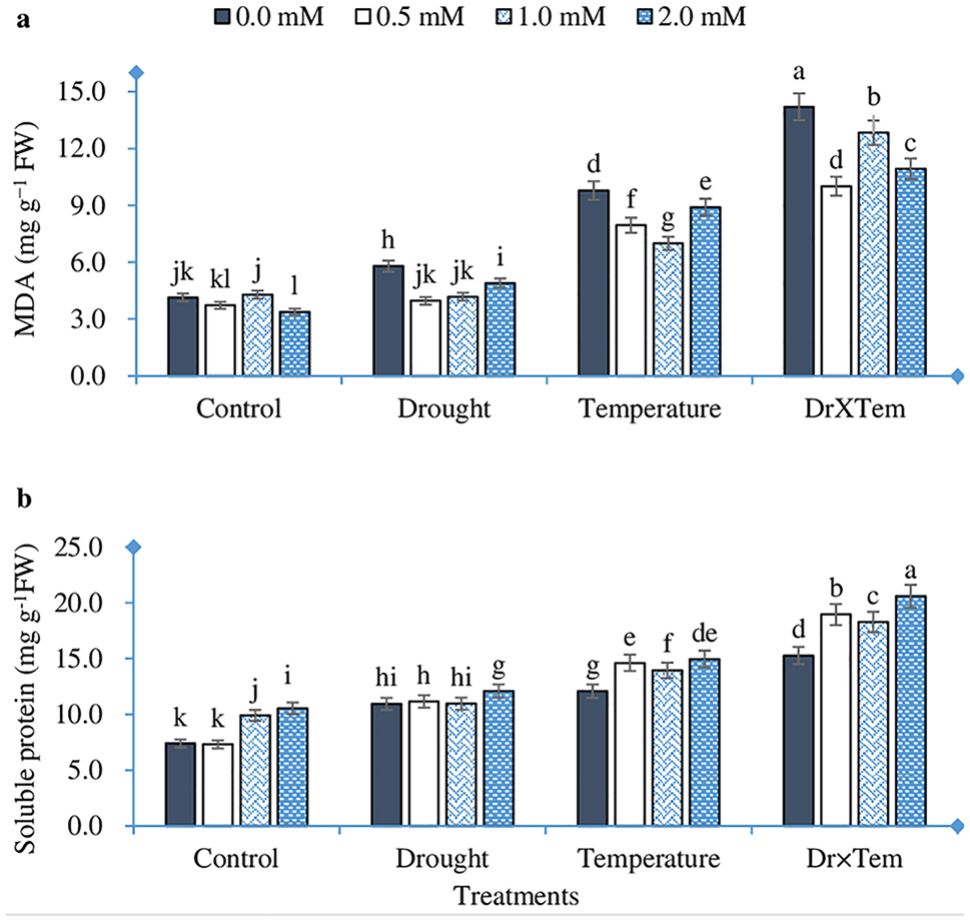
Effect of high temperature, drought, GSH, and their interaction on malondialdehyde content (**a**) and soluble protein content (**b**) of wheat seedlings. Bars labeled with different letters indicate significant difference at *P* < 0.05.

The impact of temperature, drought, GSH, and their interaction was significant for MDA and protein contents (Table 4). MDA content increased at all stress treatments, and a higher MDA content (14.20 mg g^-1^ FW) was found at combined stress. Soil pre-treatment with GSH caused a considerable reduction in MDA at all treatments. At drought and combined treatments, 0.5 mM GSH reduced MDA by 31.6 and 29.5%, respectively, compared with 0.0 mM GSH. Moreover, 1.0 mM GSH decreased MDA by 18.8% relative to 0.0 mM GSH at the temperature treatment (Fig. 2a). Both temperature and drought stress raised soluble protein content, while the combined stress increased protein content by 106.6% relative to the control. Protein content increased with increasing GSH; 2.0 mM compared with 0.0 mM GSH increased protein by 42.5, 10.5, 23.9, and 34.8% at control, drought, temperature, and the interaction, respectively (Fig. 2b).

### Antioxidant enzyme activity

**Figure 3:**
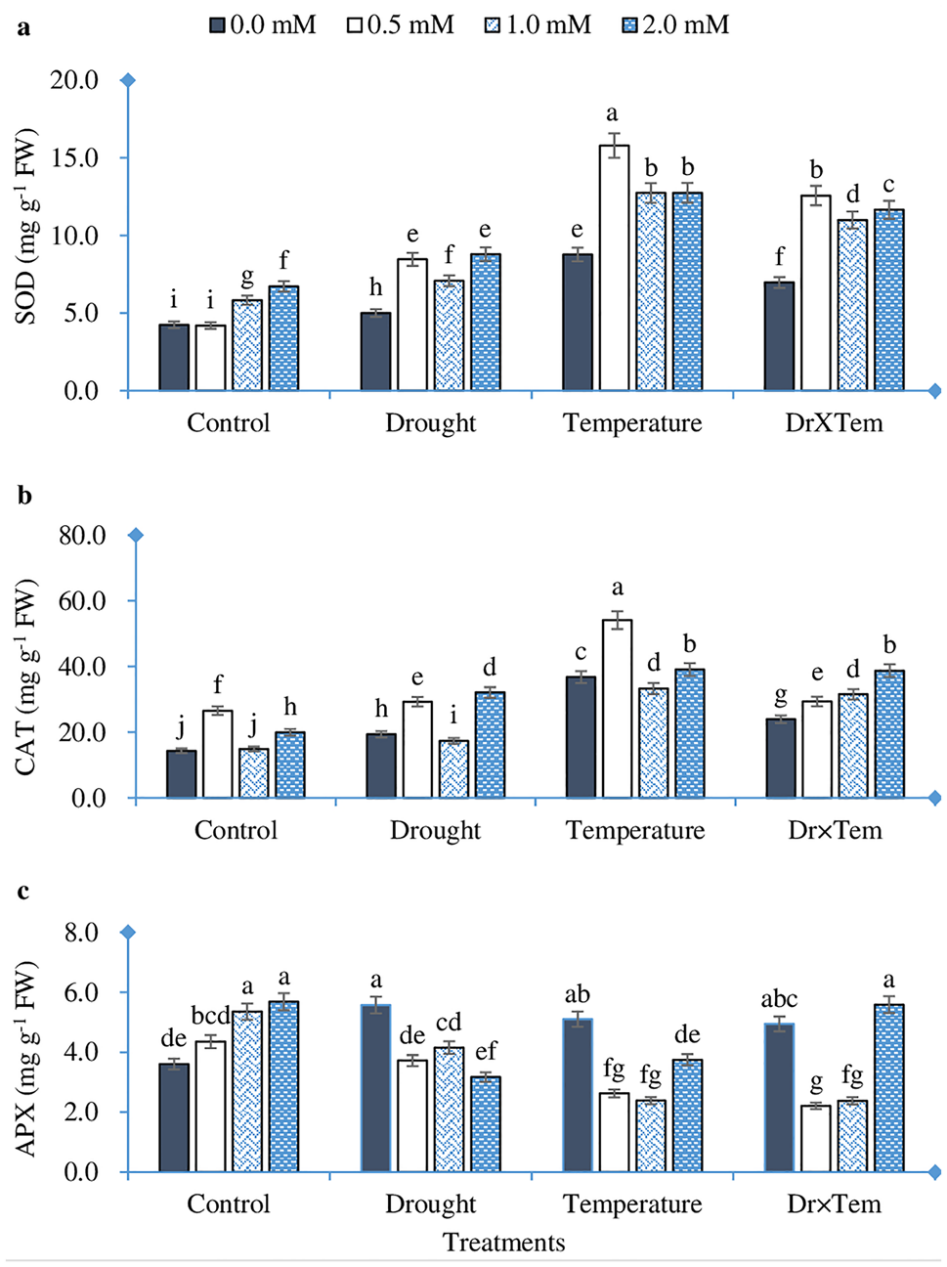
Effect of high temperature, drought, GSH, and their interaction on antioxidant enzyme activity of wheat seedling. (**a**): superoxide dismutase, (**b**): catalase, (**c**): ascorbate peroxide. Bars labeled with different letters indicate significant difference at *P* < 0.05.

As shown in Fig. 3a, b, SOD and CAT activity increased significantly in response to HT and drought stresses. However, the magnitude of the increase was much greater in HT (106.9% and 156.9%) than that in drought treatment (18.6% and 35.1%) relative to the control. However, the interaction effect increased SOD and CAT activity by 39.4% and 23.5%, respectively, compared with the control, but the increases were less than that of HT. On the other hand, GSH enhanced the activity of antioxidant enzymes. In this regard, the influence of 0.5 mM was more noticeable than other levels of GSH. Application of 0.5 mM GSH increased SOD and CAT activity by 69.3% and 51.2% in the drought treatment, respectively. Whereas, at HT treatment, they increased by 79.9% and 47.2%, respectively, relative to the control. In the combined treatments, the activity of SOD was prominently increased at 0.5 mM and then decreased, while CAT activity was increased gradually with increasing GSH doses. APX activity increased significantly in all the treatments compared to the control, where the increase in drought was much higher than that in other treatments. APX increased gradually by increasing the GSH level in the control treatment, while at the drought treatment it was reduced. Whereas, in temperature and combined stress, we found that APX was decreased at 0.5 and 1.0 mM GSH and then considerably increased at 2.0 mM (Fig. 3c).

## Discussion

HT and drought stresses are the main environmental factors that restrict crop growth and production, and their combined impact is more critical than their single effect^24^. However, the response of plants to these conditions varies at different stages of the plant's life cycle. Seedling stage is known as the most sensitive growth stage to heat and water deficit^9^. Therefore, enhancement of crop growth at the seedling stage through exogenous substances may be a feasible practice to increase crop productivity under stress conditions. In this study, HT, drought, and their combination had adverse effects on the root seedling's length, surface area, project area, volume, and forks (Table 2), with the biggest reductions in the combined treatments of HT and drought. However, root tips were raised under both stresses and more increments were recorded in the combined treatments of HT and drought. Root tip is a very useful attribute. It may increase the plant's capacity to absorb moisture from different layers of the rhizosphere. Under drought conditions, plants usually develop a better root system to respond to water scarcity. A widespread root structure is beneficial to absorb water from lower soil layers and upkeep plant growing at the early crop development stage^25^. Shoot length, leaf area, fresh and dry weights of root and shoot were decreased by HT and drought while a remarkable reduction was observed in the combined treatments (Tables 2 and 3). These results were in line with those obtained by Bachiri et al. and Suliman et al.^24,26^. Drought stress reduces soil moisture, and HT increases water loss from cells, resulting in a decline of leaf RWC and turgidity^27,28^. In this study, both HT and drought decreased RWC, while a noticeable reduction was caused by combined stress.

Application of GSH enhanced seedling growth and alleviated the adverse impact of HT and drought stresses. The exogenous or endogenous GSH level was reported to have enhanced growth under adverse environments in many plant species^22,28^. Consistent with those studies, we noted that plants treated with GSH had significantly higher shoot length, leaf area, and fresh and dry weights of root and shoot than the control plants (Tables 2 and 3). Ding et al.^22^ reported that GSH promoted physiological and morphological adaptation to heat stress by modulating the photosynthesis, antioxidant, and osmolyte systems. Furthermore, seedlings treated with GSH improved root traits (Table 2) and thus retained soil water uptake. It may be one of the main factors that motivates the highest level of the leaf RWC in the treated plants (Table 3). Our results agree with those of Pei et al.^28^. Maintenance of leaf water status is a critical point for plants facing heat and water scarcity. Water is responsible for physiological and biochemical reactions in the plant cells, which is used as the criterion for recognizing plants with contrasting variances in water deficit tolerance^29^. The content of osmoprotectants can increase under stress conditions and reveal water shortage through osmotic adjustment. Accumulation of these organic molecules in the tissue promotes water retention in the plant. Soluble protein, known as an osmoprotectant, is reported to increase under heat and drought stresses in many plants^26,30^. Our study showed that GSH significantly raised protein content in HT and/or drought-influenced wheat seedlings (Fig. 2b), which might be another factor to enhance leaf RWC.

Photosynthesis is one of the main biological processes that is very sensitive to HT stress and is often inhibited before other cell functions are impaired^31^. This process mainly depends on photosynthetic pigments, including chlorophyll *a*, chlorophyll *b*, and total chlorophyll contents. In this study, HT, drought, and their combinations imposed a significant negative impact on these variables (Fig. 1). This result agrees with previous findings of Nahar et al.^32^. The reduction in chlorophyll contents in wheat seedlings may be due to the damage of chloroplast structure^33^, which occurs when plants are subjected to adverse environmental conditions including HT and drought. GSH is located in the chloroplasts and other parts of the plant, but 80% of GSH activity in photosynthetic tissue occurs by the chloroplast isoform^34^. Therefore, GSH could have a role in alleviating injuries to photosynthetic pigments and chloroplast membranes induced by stress conditions, which might explain the higher chlorophyll *a*, chlorophyll *b*, and total chlorophyll contents of seedlings treated with GSH and subjected to HT and/or drought (Fig. 1). Our results are in line with those of Ding et al.^22^.

Environmental stress like HT and drought adversely affects physiological and biochemical activities. Such extreme stresses lead to an imbalance between the light reactions and the Calvin–Benson cycle, which over-reduces the electron carriers in chloroplasts and mitochondria, resulting in ROS production^35^. Lipid peroxidation is the most determined consequence of reactive oxygen species activity on the function and structure of cell membrane content^36^. MDA content is a product of lipid peroxidation under stress conditions. In this study, HT and drought stress exhibited a significant increase in the MDA content of wheat seedlings, and more severe damage effects were observed when both factors were applied (Fig. 2a). This result corroborates with other results obtained by Nahar et al.^32^, who attributed the increase in MDA to the fact that HT stimulates the generation and reactions of ROS, thereby inducing oxidative stress. However, wheat seedlings treated with GSH significantly reduced MDA content (Fig. 2a). Similar results were obtained by Nahar et al.^17^ in mung bean seedlings under HT. Endogenous GSH was detected to reduce oxidative stress in the plants under various abiotic stresses^37,38^. GSH pre-treatment induced enhancement in cell membrane properties and increased the endogenous level of GSH content^17^, which consequently improved lipid peroxidation stability and seedling tolerance to HT and drought stress.

Previous studies indicated that heat and drought stress increased O_2_^-^, H_2_O_2_, and MDA in wheat and other plant species^26,32,39,40^. Stressed plants can stimulate self-mechanisms to scavenge oxidative products to a certain level. One of these mechanisms is the ability of plants to maintain higher activity of the antioxidant enzyme system, which includes SOD, CAT, and APX^24^. Antioxidant enzymes play an important role in scavenging O_2_^-^ and H_2_O_2_, catalyzing their conversion to H_2_O and O_2_^41^. In the present study, HT and/or drought stresses induced increases in SOD and CAT and considerable increases when GSH was applied (Fig. 3a,b). APX participated in H_2_O_2_ scavenging by converting it to H_2_O through the AsA cycle^17,42^. In our study, APX significantly increased under both the single and combined stress of HT and drought. Exogenous GSH increased APX activity at the control and combined treatments, while the activity was decreased at the individual treatment of HT and drought (Fig. 3c). In addition to its role in promoting the activity of the enzymes mentioned above. GSH is involved in the AsA-GSH cycle and can be regenerate other potential water-soluble antioxidants through the AsA-GSH cycle, which acts as a substrate for several antioxidant enzyme systems such as GPX and GST^17,18^. As the AsA-GSH cycle is fully present in chloroplasts and functional in plant mitochondria to neutralise H_2_O_2_^43^. Previous studies also confirmed the valuable role of GSH in up-regulation of plant defence mechanisms under abiotic stresses^17,44,45^.

## Conclusions

This study demonstrated that HT and drought stress-induced growth inhibition of wheat seedlings and magnitude inhibition occurred when both factors were applied. The growth reduction is associated with increased MDA and deterioration of the chlorophyll pigments. GSH has a vital role in improving wheat seedlings' resistance to oxidative stress damage. Its role was involved in enhancing the root system, dry matter accumulation, and leaf relative water content. Furthermore, GSH also reduced MDA content, increased chlorophyll pigments, and antioxidant enzyme activity. 0.5 mM showed better performance among different GSH concentrations. Therefore, GSH can be applied appropriately to increase the HT and/or drought tolerance of wheat seedlings by regulating physiological attributes.

## Materials and methods

### Ethics statement

The authors confirm that all methods were carried out in accordance with the relevant guidelines and regulations.

### Procedures

A controlled study was conducted in 2021 at the Joint International Research Laboratory of Agriculture and Agri-Product Safety of the Ministry of Education of China, Yangzhou University, (32.30° N, 119.43° E). The seeds of wheat variety Gomria, obtained from the Ministry of Agriculture of Sudan, were used. Fifteen seeds with uniform size were sown in each plastic pot (9.5 cm in diameter, 8.5 cm in depth, and without holes at the bottom) filled with 400 g washed and sterilized sands. The study was designed as a 3-factorial experiment, arranged in a completely randomized design with three replications for each treatment. This experiment was conducted twice, and the average of the means was presented. The experiment was divided into two sets according to temperature levels. One set as a control was grown at 25 °C, and the other one as heat stress was subjected to a high temperature (HT) of 33 °C lasting for six days. The two growth chambers were set at 55–60% RH and photoactive radiation of 500 W m^-2^ (12/12 h day/night). Ninety ml of four levels (0.0, 0.5, 1.0, and 2.0 mM) of glutathione solution were added to the soil after the 4th, 7th, and 10th days of seeding. On the 8th day after sowing, two water levels were applied, i.e., normal watering (80 ml pot^-1^) and water deficit (60% of normal watering), designed as W_n_ and W_d_, respectively, were applied at a 3-day interval based on a preliminary test on soil field capacity, and also based on our previous study. During the study period, 150 ml of full-strength Hoagland solution was added to each pot on the 1st, 8th, and 10th days after seed sowing.

### Measurements

#### Growth attributes

Two weeks after sowing, all the seedlings were harvested. Five seedlings from each pot were selected randomly to measure leaf area, shoot length, fresh weight, and dry weight of root and shoot. Leaf area was measured by using a portable Area Meter device (LI-3100C, LI. COR. inc. Lincoln, Nebraska, USA). The shoot length was determined by using a ruler. The shoots and roots of plants were weighed and dried in an oven at 70℃ for 72 H to a constant weight for dry weight determination. The roots of the three plants were washed with deionized water. Imagery scan screen (12,000 XL, Seko Epson CO. Ltd, Japan) was used to perform root scanning. Further, WinRHIZO software was used for root image analysis.

### Leaf relative water content (RWC)

RWC was determined according to the method of Mäkelä et al.^46^. Leaf samples were collected from the studied plants. The fresh weight (FW) was measured, and the samples were kept in water for 4 h for saturation weight (FWsat) determination. The samples were dried in a hot air oven at 80 °C for 48 h to determine the dry weight (DW).

The RWC was calculated as follows:

$${\text{RWC }}\left( \% \right) = \left[ {\left( {{\text{FW}} - {\text{DW}}} \right)/\left( {{\text{FWsat}} - {\text{DW}}} \right)} \right] \times {\text{1}}00$$

### Physiological attributes

#### Chlorophyll *a* and *b* total chlorophyll content

Leaf supernatant was extracted with 80% v/v acetone. The absorbance reading was measured with a UV–visible spectrophotometer (Model A360, AOE Inc, Shanghai, China) at 663 and 645 nm, and chlorophyll content was calculated according to Lichtenthaler and Wellburn^47^.

### Lipid peroxidation

The level of lipid peroxidation was measured by assessing malondialdehyde (MDA) content according to Zhang et al.^48^.

### Soluble protein determination

The protein concentration of each sample was determined following the method of Bradford^49^, using bovine serum albumin as a protein standard.

### Enzyme extraction and assays

Leaves, collected and ground into fine powder in liquid nitrogen with a mortar and pestle, were homogenized at 4 °C in 2 ml in 50 mM of phosphate-buffered solution (pH 7.8), 0.1 mM EDTA, 0.3% TritonX-100, 4% polyvinylpolypyrroidone; and centrifuged at 4 °C, 10,500 rpm, for 20 min. The supernatant was used to assay antioxidant enzymes activity. The activities of SOD, CAT, and APX were determined according to the methods of Koca et al.^50^, Raza et al.^51^, and Nakano and Asada^52^, respectively.

### Statistical analyses

This study was a 3-factorial design arranged in a completely randomized design with three replicates for each treatment. The data collected were subjected to ANOVA with the statistical package of MSTAT-C^53^. When F values were significant, means were separated by the least significant differences (LSD) test at the 0.05 probability level.

### Ethics statement

The authors confirm that all methods were carried out in accordance with the relevant guidelines and regulations.

## Data Availability

All data generated and analyzed in this study are included in this manuscript.

## References

[1] Iqbal MA, Hussain I, Siddiqui MH, Ali E, Ahmad Z (2018). Probing profitability of irrigated and rainfed bread wheat (*Triticum aestivum* L.) crops under foliage applied sorghum and moringa extracts in Pakistan. Cust. e Agron.

[2] Shaukat M (2021). Genetic gain for grain micronutrients and their association with phenology in historical wheat cultivars released between 1911 and 2016 in Pakistan. Agronomy.

[3] Jahan M (2019). Effect of naphthaleneacetic acid on root and plant growth and yield of ten irrigated wheat genotypes. Pak. J. Bot..

[4] Tadesse W (2019). Genetic gains in wheat breeding and its role in feeding the world. Crop Breed. Genet. Genom.

[5] Mahrookashani A, Siebert S, Hüging H, Ewert F (2017). Independent and combined effects of high temperature and drought stress around anthesis on wheat. J. Agron. Crop Sci..

[6] Sareen S (2023). Resilience to terminal drought, heat, and their combination stress in wheat genotypes. Agronomy.

[7] Nawaz F (2021). Pretreatment with selenium and zinc modulates physiological indices and antioxidant machinery to improve drought tolerance in maize (*Zea mays* L.). S. Afr. J. Bot..

[8] Djanaguiraman M, Boyle D, Welti R, Jagadish S, Prasad P (2018). Decreased photosynthetic rate under high temperature in wheat is due to lipid desaturation, oxidation, acylation, and damage of organelles. BMC Plant Biol..

[9] Ali S (2020). Approaches in enhancing thermotolerance in plants: an updated review. J. Plant Growth Regul..

[10] Hossain A (2021). Consequences and mitigation strategies of abiotic stresses in wheat (*Triticum aestivum* L.) under the changing climate. Agronomy.

[11] Wang G-P (2010). Improvement of heat and drought photosynthetic tolerance in wheat by overaccumulation of glycinebetaine. Plant Biotechnol. Rep..

[12] Azmat A (2022). Coactive role of zinc oxide nanoparticles and plant growth promoting rhizobacteria for mitigation of synchronized effects of heat and drought stress in wheat plants. Chemosphere.

[13] Baxter A, Mittler R, Suzuki N (2014). ROS as key players in plant stress signalling. J. Exp. Bot..

[14] Yousuf, P. Y., Hakeem, K. U. R., Chandna, R. & Ahmad, P. in *Abiotic Stress Responses in Plants* 149–158 (Springer, 2012).

[15] Zagorchev L, Seal CE, Kranner I, Odjakova M (2013). A central role for thiols in plant tolerance to abiotic stress. Int. J. Mol. Sci..

[16] Foyer CH, Noctor G (2003). Redox sensing and signalling associated with reactive oxygen in chloroplasts, peroxisomes and mitochondria. Physiologia Plantarum.

[17] Nahar K, Hasanuzzaman M, Alam MM, Fujita M (2015). Exogenous glutathione confers high temperature stress tolerance in mung bean (*Vigna radiata* L.) by modulating antioxidant defense and methylglyoxal detoxification system. Environ. Exp. Bot..

[18] Szalai G, Kellős T, Galiba G, Kocsy G (2009). Glutathione as an antioxidant and regulatory molecule in plants under abiotic stress conditions. J. Plant Growth Regul..

[19] Foyer CH, Noctor G (2005). Redox homeostasis and antioxidant signaling: a metabolic interface between stress perception and physiological responses. Plant Cell.

[20] Cao, F., Fu, M., Wang, R., Diaz-Vivancos, P. & Hossain, M. A. in *Glutathione in Plant Growth, Development, and Stress Tolerance* 171–194 (Springer, 2017).

[21] Nahar K, Hasanuzzaman M, Alam M, Fujita M (2015). Glutathione-induced drought stress tolerance in mung bean: Coordinated roles of the antioxidant defence and methylglyoxal detoxification systems. AoB Plants.

[22] Ding X (2016). Exogenous glutathione improves high root-zone temperature tolerance by modulating photosynthesis, antioxidant and osmolytes systems in cucumber seedlings. Sci. Rep..

[23] Akram S (2017). Exogenous glutathione modulates salinity tolerance of soybean [*Glycine max* (L.) Merrill] at reproductive stage. J. Plant Growth Regul..

[24] Bachiri H, Djebbar R, Mekliche A (2020). The effect of drought, heat and combined stress on antioxidant enzymes in bread wheat genotypes (*Triticum Aestivum* L.). Analele Universităţii din Oradea, Fascicula Biologie.

[25] Hasanuzzaman, M. *Agronomic crops: volume 3: Stress responses and tolerance*. (Springer Nature, 2020).

[26] Suliman MSE (2021). Foliar application of 5-aminolevulinic acid alleviated high temperature and drought stresses on wheat plants at seedling stage. Chil. J. Agric. Res..

[27] Zeng W, Hassan MJ, Kang D, Peng Y, Li Z (2021). Photosynthetic maintenance and heat shock protein accumulation relating to γ-aminobutyric acid (GABA)-regulated heat tolerance in creeping bentgrass (*Agrostis stolonifera*). S. Afr. J. Bot..

[28] Pei L (2019). Role of exogenous glutathione in alleviating abiotic stress in maize (*Zea mays* L.). J. Plant Growth Regul..

[29] Zhou S (2012). Overexpression of the wheat aquaporin gene, TaAQP7, enhances drought tolerance in transgenic tobacco. PloS one.

[30] Liu S, Waqas MA, Wang S-H, Xiong X-Y, Wan Y-F (2017). Effects of increased levels of atmospheric CO_2_ and high temperatures on rice growth and quality. PloS one.

[31] Mathur S, Agrawal D, Jajoo A (2014). Photosynthesis: Response to high temperature stress. J. Photochem. Photobiol. B: Biol..

[32] Nahar K (2017). Insights into spermine-induced combined high temperature and drought tolerance in mung bean: Osmoregulation and roles of antioxidant and glyoxalase system. Protoplasma.

[33] Khan N (2017). Effect of heat stress on growth, physiological and biochemical activities of wheat (*Triticum aestivum* L.). Int. J. Biosci..

[34] Edwards EA, Rawsthorne S, Mullineaux PM (1990). Subcellular distribution of multiple forms of glutathione reductase in leaves of pea (*Pisum sativum* L.). Planta.

[35] Foyer CH, Noctor G (2012). Managing the cellular redox hub in photosynthetic organisms. Plant, Cell Environ..

[36] Puyang X, An M, Han L, Zhang X (2015). Protective effect of spermidine on salt stress induced oxidative damage in two Kentucky bluegrass (*Poa pratensis* L.) cultivars. Ecotoxicol. Environ. Saf..

[37] Wu J, Sun S, Ke Y, Xie C, Chen F (2010). Effects of glutathione on chloroplast membrane fluidity and the glutathione circulation system in young loquat fruits under low temperature stress. Acta Hortic..

[38] Hasanuzzaman, M. *et al.* Exogenous proline and glycine betaine mediated upregulation of antioxidant defense and glyoxalase systems provides better protection against salt-induced oxidative stress in two rice (*Oryza sativa* L.) varieties. *BioMed Res. Int.*, 757219 (2014). 10.1155/2014/757219 PMC4065706 24991566

[39] Khushboo (2018). Exogenous application of calcium chloride in wheat genotypes alleviates negative effect of drought stress by modulating antioxidant machinery and enhanced osmolyte accumulation. In Vitro Cell. Develop. Biol.-Plant.

[40] Liu J (2019). High temperature and drought stress cause abscisic acid and reactive oxygen species accumulation and suppress seed germination growth in rice. Protoplasma.

[41] Anwar A, Wang J, Yu X, He C, Li Y (2020). Substrate Application of 5-aminolevulinic acid enhanced low-temperature and weak-light stress tolerance in cucumber (*Cucumis sativus* L.). Agronomy.

[42] Wei-Feng X, Wei-Ming S, Ueda A, Takabe T (2008). Mechanisms of salt tolerance in transgenic Arabidopsis thaliana carrying a peroxisomal ascorbate peroxidase gene from barley. Pedosphere.

[43] Miller G, Suzuki N, Ciftci-Yilmaz S, Mittler R (2010). Reactive oxygen species homeostasis and signalling during drought and salinity stresses. Plant, Cell Environ..

[44] Yu C-W, Murphy TM, Lin C-H (2003). Hydrogen peroxide-induced chilling tolerance in mung beans mediated through ABA-independent glutathione accumulation. Funct. Plant Biol..

[45] Daud M (2016). Leaf-based physiological, metabolic, and ultrastructural changes in cultivated cotton cultivars under cadmium stress mediated by glutathione. Environ. Sci. Pollut. Res..

[46] Mäkelä P, Munns R, Colmer T, Condon A, Peltonen-Sainio P (1998). Effect of foliar applications of glycinebetaine on stomatal conductance, abscisic acid and solute concentrations in leaves of salt-or drought-stressed tomato. Funct. Plant Biol..

[47] Lichtenthaler HK, Wellburn AR (1983). Determinations of total carotenoids and chlorophylls *a* and *b* of leaf extracts in different solvents. Biochem. Soc. Transact..

[48] Zhang S, Hu J, Zhang Y, Xie X, Knapp A (2007). Seed priming with brassinolide improves lucerne (*Medicago sativa* L.) seed germination and seedling growth in relation to physiological changes under salinity stress. Aust. J. Agric. Res..

[49] Bradford N (1976). A rapid and sensitive method for the quantitation microgram quantities of a protein isolated from red cell membranes. Anal. Biochem..

[50] Koca H, Bor M, Özdemir F, Türkan İ (2007). The effect of salt stress on lipid peroxidation, antioxidative enzymes and proline content of sesame cultivars. Environ. Exp. Bot..

[51] Raza SH, Athar HR, Ashraf M, Hameed A (2007). Glycinebetaine-induced modulation of antioxidant enzymes activities and ion accumulation in two wheat cultivars differing in salt tolerance. Environ. Exp. Bot..

[52] Nakano Y, Asada K (1981). Hydrogen peroxide is scavenged by ascorbate-specific peroxidase in spinach chloroplasts. Plant Cell Physiol..

[53] Freed R (1991). MSTAT-C: A microcomputer program for the design, management, and analysis of agronomic research experiments.

