# Author Correction: Exogenous glutathione protected wheat seedling from high temperature and water deficit damages

**DOI:** 10.1038/s41598-024-57808-2

**Published:** 2024-03-26

**Authors:** Mohamed Eltyeb Suliman Suliman, Safiya Babiker Mustafa Elradi, Guisheng Zhou, Tianyao Meng, Guanglong Zhu, Yunji Xu, Nimir Eltyb Ahmed Nimir, Aboagla Mohammed Ibrahim Elsiddig, Atef Hemaida Mohammed Awdelseid, Adam Yousif Adam Ali, Xiaoqian Guo, Irshad Ahmad

**Affiliations:** 1 Joint International Research Laboratory of Agriculture and Agri-Product Safety of the Ministry of Education of China, , Yangzhou University, Yangzhou, 225009 China; 2 Faculty of Agriculture, University of Khartoum, 13314 Shambat, Khartoum, Sudan; 3 Jiangsu Co-Innovation Center for Modern Production Technology of Grain Crops, Yangzhou University, Yangzhou, 225009 China; 4 Department of Agronomy, College of Agricultural and Environment Science, University of Al Qadarif, 32214 Al Qadarif, Sudan

Correction to: *Scientific Reports * 10.1038/s41598-023-47868-1, published online 04 March 2023

The Acknowledgments section in the original version of this Article was incomplete.

“This research was financially supported by China National Key R & D Program (2022YFE0113400) and Jiangsu Provincial Fund for Realizing Carbon Emission Peaking and Neutralization (BE2022305).”

now reads:

“This research was financially supported by China National Key R & D Program (2022YFE0113400) and Jiangsu Provincial Fund for Realizing Carbon Emission Peaking and Neutralization (BE2022305). “Belt and Road” innovation talent exchange for foreign experts program of the Ministry of Science and Technology (DL2023014011L).”

The original Article has been corrected.

